# Decentralized solar-powered drinking water ozonation in Western Kenya: an evaluation of disinfection efficacy

**DOI:** 10.12688/gatesopenres.13138.2

**Published:** 2020-10-15

**Authors:** Colin Hendrickson, Jared Oremo, Oscar Oluoch Akello, Simon Bunde, Isaac Rayola, David Akello, Daniel Akwiri, Sung-Jin Park, Samuel Dorevitch

**Affiliations:** 1 School of Public Health, University of Illinois at Chicago, Chicago, IL, 60612, USA; 2 Safe Water and AIDS Project (SWAP), Kisumu, 3323-40100, Kenya; 3 Shemjen Engineering, Nairobi, 23697-00100, Kenya; 4 Electrical and Computer Engineering, University of Illinois at Urbana-Champaign, Urbana, IL, 61801, USA; 5 Institute for Environmental Science and Policy, University of Illinois at Chicago, Chicago, IL, 60612, USA

**Keywords:** Decentralized water treatment, drinking water treatment, ozonation, solar-powered water treatment, water quality, sustainable water treatment, microplasma technology

## Abstract

** Background:** Decentralized drinking water treatment methods generally apply membrane-based treatment approaches. Ozonation of drinking water, which previously has only been possible at large centralized facilities, can now be accomplished on a small-scale using microplasma technology. The efficacy of decentralized solar-powered drinking water treatment systems has not previously been described.

**Methods:** We established a 1,000L decentralized solar-powered water treatment system located in Kisumu County, Kenya. Highly contaminated surface water is pumped to the treatment system, which includes flocculation and filtration steps prior to ozonation. Turbidity, total coliform bacteria, and *E. coli *were measured at various stages of water treatment, and bacterial log reduction values (LRVs) were calculated.

**Results:** Forty-seven trials were conducted in which1000L of water were flocculated, filtered, and ozonated for 180 minutes. Baseline turbidity and *E. coli* concentrations were reduced from a median of 238 nephelometric turbidity units (NTU) and 2,419.7 most probable number/100mL, respectively, in surface water to 1.0 NTU and undetectable *E. coli* after ozonation for 180 minutes. The median *E. coli *LRV was 3.99.

**Conclusions:** The solar-powered, decentralized water treatment system that utilizes ozonation for disinfection was founded to reduce *E. coli* by more than 3 log-orders of magnitude despite the high turbidity of the raw water. Further research is needed to characterize limitations, scalability, economic viability, and community perspectives that could help determine the role for similar systems in other settings.

## Abbreviations

AC, Alternating current; CFU, colony forming units; DC, Direct current; Hz, Hertz; g, grams; mL, Milliliters; L, Liter; LRV, Log reduction value; MPN, Most probable number; ND, non-detect; NTU, Nephelometric turbidity units; POU, Point of use; USEPA, United States Environmental Protection Agency; V, Volt; W, Watt; WHO, World Health Organization

## Introduction

In 2017, an estimated 357 million cases of diarrhea occurred among children under the age of five years in low- and middle-sociodemographic index countries, resulting in approximately 222,457 deaths ( IHME, 2020). Of those deaths, 93.5% are attributable to unsafe water, sanitation, and handwashing. Meeting Target 6.1 of the United Nations Sustainable Development Goals - universal and equitable access to safe and affordable drinking water by 2030 - should therefore substantially reduce the global burden of waterborne diarrheal disease in children. Meeting that target will require innovative and effective interventions, as an estimated 144 million people, more than half of whom live in Sub-Saharan Africa, collect drinking water directly from surface waters ( UNICEF & World Health Organization, 2019).

The costs of constructing centralized drinking water treatment systems and water distribution systems, as well as the costs for operations and maintenance of such systems, are in the hundreds of millions of dollars ( Plappally & Lienhard, 2013), far beyond the reach of many low- and middle-income countries. Point-of-use (POU) water treatment has been utilized as an alternative approach to centralized treatment in such settings. While certainly far less costly, the long-term adherence to POU water treatment is highly variable and often poor ( Roma *et al.*, 2014; Rosa & Clasen, 2017; Rothstein *et al.*, 2015), perhaps in part due to challenges in adherence. High-quality intervention studies have not reported consistent reductions of diarrhea occurrence in children in association with POU water treatments ( Clasen *et al.*, 2015).

Another type of alternative to centralized drinking water treatment is decentralized treatment ( Peter-Varbanets *et al.*, 2009). Such systems produce water, typically using membrane-based ultrafiltration with or without chlorination or ultraviolet disinfection. At decentralized treatment stations, community members can refill containers - generally approximately 20L – with treated water. Refill kiosks at decentralized systems have become local businesses in both urban and rural areas of Southeast Asia ( Sima & Elimelech, 2013). In an evaluation of 10 decentralized membrane-based systems in rural healthcare facilities in rural Rwanda, the systems were found to consistently provide high-quality water without the need for technical expertise to operate ( Huttinger *et al.*, 2015). However, the use of those systems was limited to areas with robust access to water and electricity.

A strategy that has not be explored is to bring decentralized systems to areas without reliable access to electricity by deploying treatment systems that run solely on solar energy. New microplasma technology has made it possible to produce ozone using small, scalable units, with three times the efficiency of conventional dielectric barrier discharge or corona ozone reactors ( Kim *et al.*, 2017; Simek & Clupek, 2002). We recently evaluated that technology as a POU water treatment in Kisian, Kenya ( Dorevitch *et al.*, 2020). Ten families were asked to ozonate their household stored water in 20L containers for a two-hour period before using the water for drinking. Water quality was monitored weekly for eight weeks. The median (10 ^th^, 90 ^th^ percentile) concentration of *E. coli* in household-stored water was 203.7 (7.9, 2,419.7) most probable number (MPN) per 100mL water. Household drinking water (which was meant to have been ozonated) had median *E. coli* concentrations of 11.4 (0.9, 369.7) MPN/100L. Thus, while *E. coli* levels decreased as a result of ozonation, they were generally above safe levels (i.e., zero), perhaps in part due to the high median turbidity of household-stored water of 48.6 nephelometric turbidity units (NTU). While solar-powered ozonation showed promise as a POU method, the deployment of the technology would be more efficient if several ozone generators simultaneously disinfected larger volumes of water over longer periods of time. The addition of turbidity reduction steps would be expected to increase the efficacy of the disinfection process. 

The primary goals of this research are to: 1) describe what we believe is the first decentralized solar-powered drinking water treatment system using ozone disinfection, and 2) to evaluate the performance of that system in treating surface water.

## Methods

### Materials

**Figure 1.**
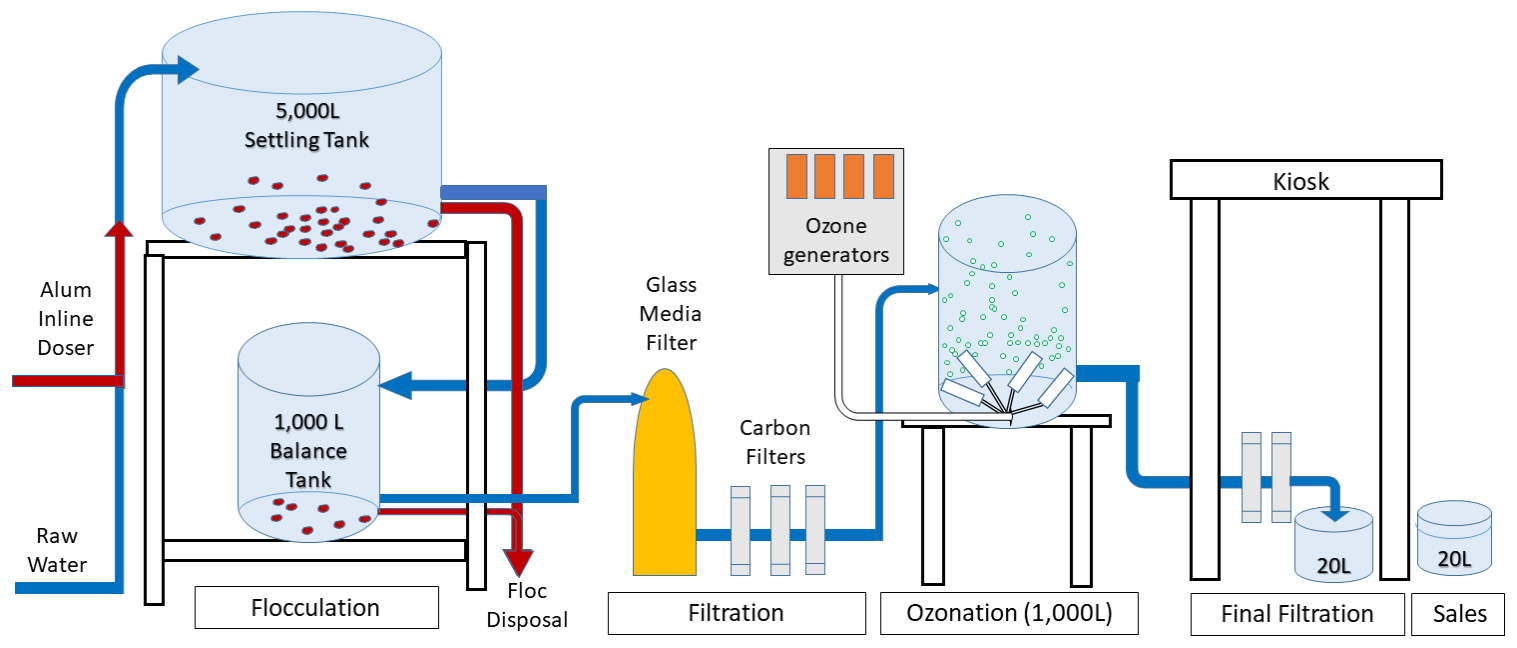
Schematic diagram of the water treatment system.

**Figure 2.**
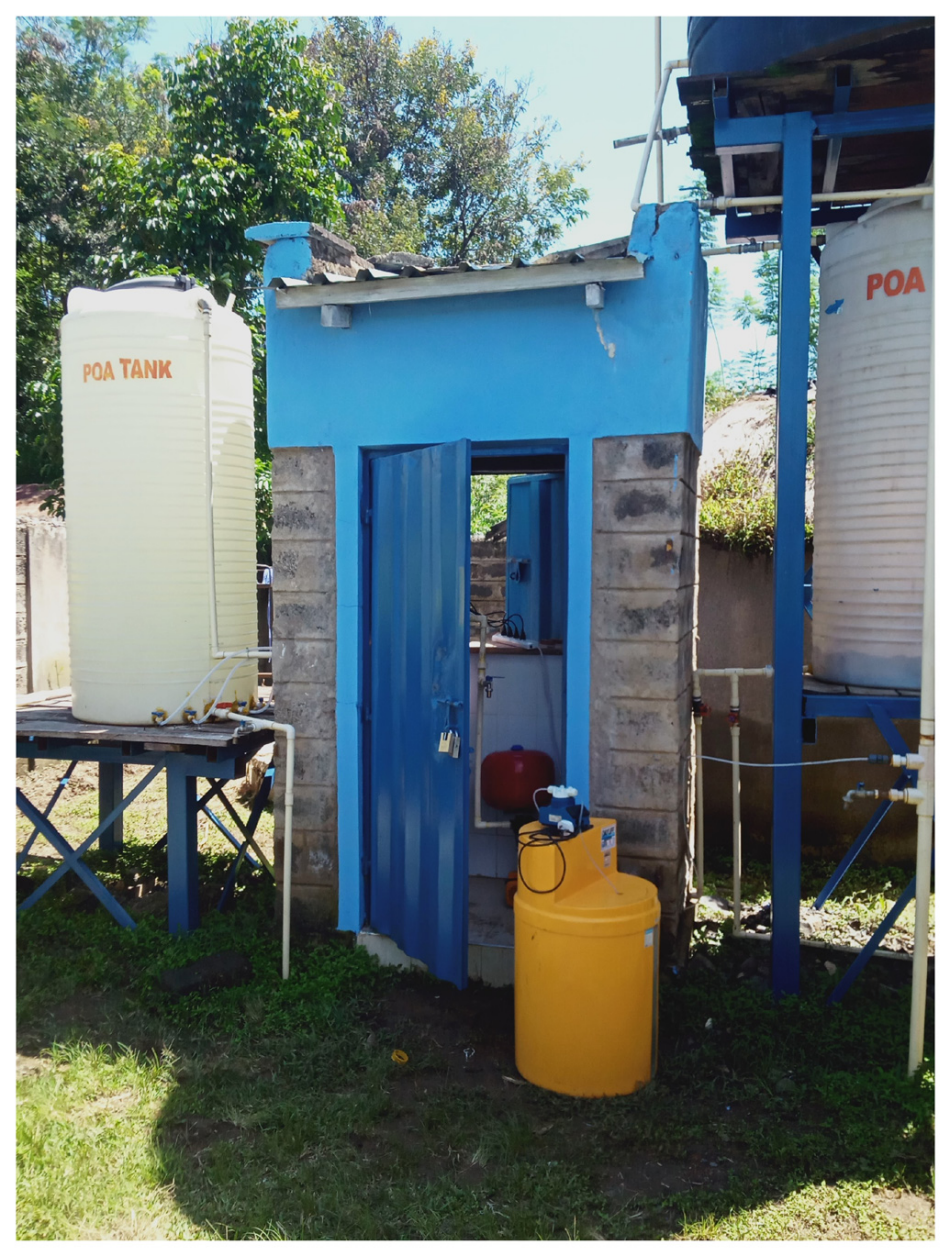
Utility housing. Upper right: The large black tank on the right is the settling tank; Right: The white tank is the balance tank; Left: The white tank is the ozonation tank. Tubing carrying ozone to the diffusers (not visible) can be seen entering the tank. Yellow container in the foreground contains alum.

**Figure 3.**
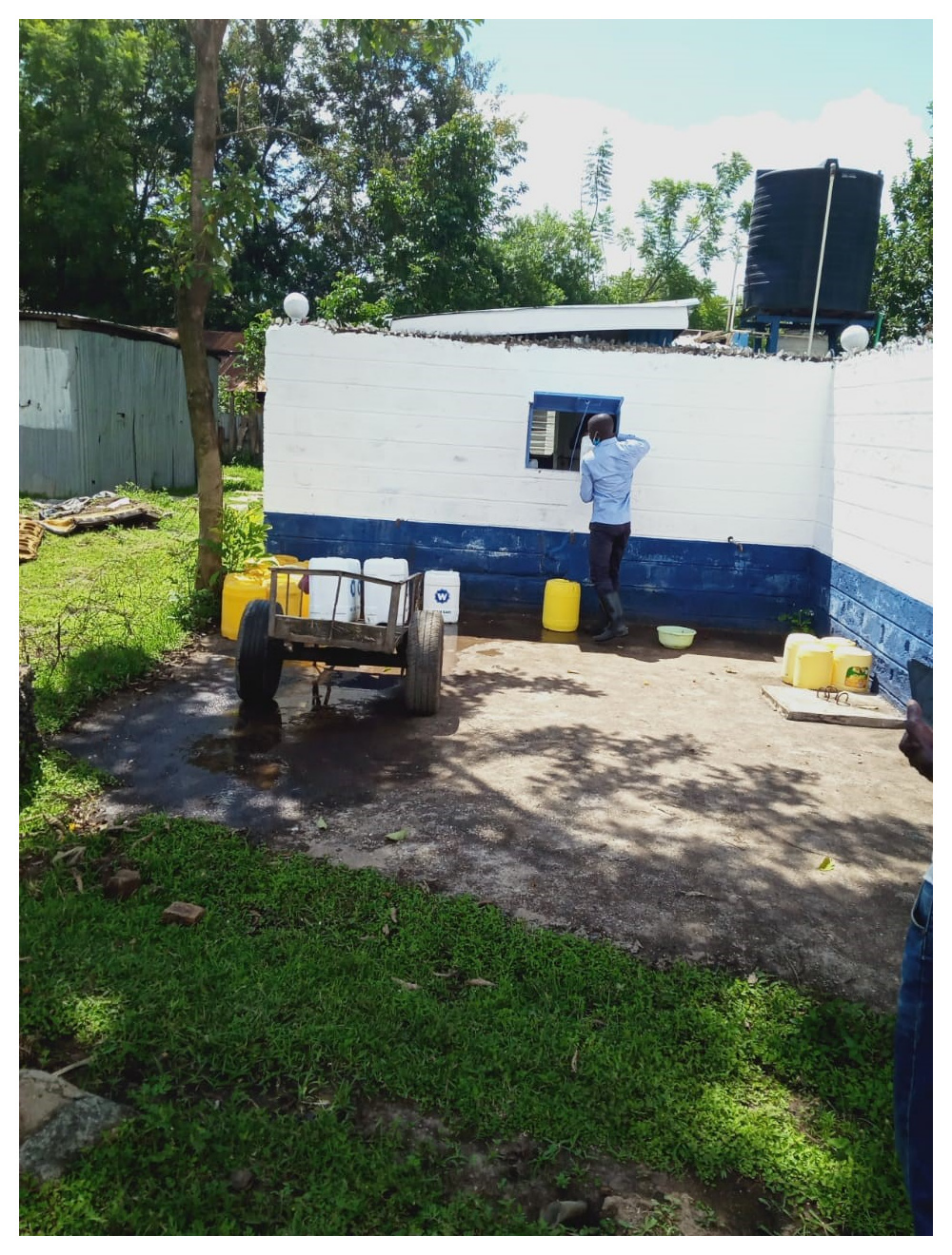
Sales kiosk. Jerrycans of water being prepared by kiosk staff for loading onto a water vendor’s cart. The black tank on the upper right is the settling tank.

***Water treatment system.*** A schematic diagram ( Figure 1) identifies key elements of the disinfection process. The three tanks and the housing for the ozone generator and initial (pre-ozonation) filters occupy an area of approximately 3 × 5 m. Five thousand L of river water were pumped from the River Nyando by a Pedrollo PKm100 1.1KW Pump over a period of approximately 1 hour. The raw water flowed through a PVC pipe and just before the 5,000L settling tank, an inline dosing pump (22W Grundfos DDC 6–10 Dosing Pump (240V) injected the water with the flocculating agent alum (powder aluminum sulfate; Kurita Water Industries) from a 100L fiberglass tank. The alum dose desired was 1g/L based on trials of flocculating highly turbid water ( Preston *et al.*, 2010); however the specific container used in preliminary field trials of ozonation held 935g, and that remained the mass utilized once the dosing system was automated. After suspended solids settled overnight, water was released from the settling tank and flowed by gravity to the balance tank through an outlet several cm above the bottom of the tank. Settled floc is removed via a clear-out valve below the outlet to the balance tank. Additional settling of solids took place in the balance tank. A 240V, 0.5 horsepower electric pump (Electric Filter (Pedrollo Linz Pump) moved water from the balance tank into a rapid glass filter containing Certikin glass media (3mm) and Jacobbi Activated carbon media chippings. Glass media is generally less costly than sand and in a laboratory evaluation of sand and glass filtration, was found to provide more rapid filtration without a loss of efficacy ( Healy *et al.*, 2010). Water was then pumped approximately 1.5 meters in elevation into the 1,000L PVC ozonation tank. The process of water filtration and filling of the ozonation tank took approximately 45 minutes. Following ozonation (described next), the water flows through the So-Safe Triple Multibody 10” Filter (8 bar) with an activated carbon and two sediment cartridges. After that step, the filtered water is considered to be “finished water.” A 5,000L tank collects rooftop harvested rainwater, so that if, during wet weather, cloud cover is too heavy to power the system (this has yet to occur), rainwater can be pumped into the ozonation tank. A utility housing ( Figure 2) contained the filters, pumps, and ozone generators. Finished water was available to the public and water vendors at the on-site water kiosk ( Figure 3).

**Figure 4.**
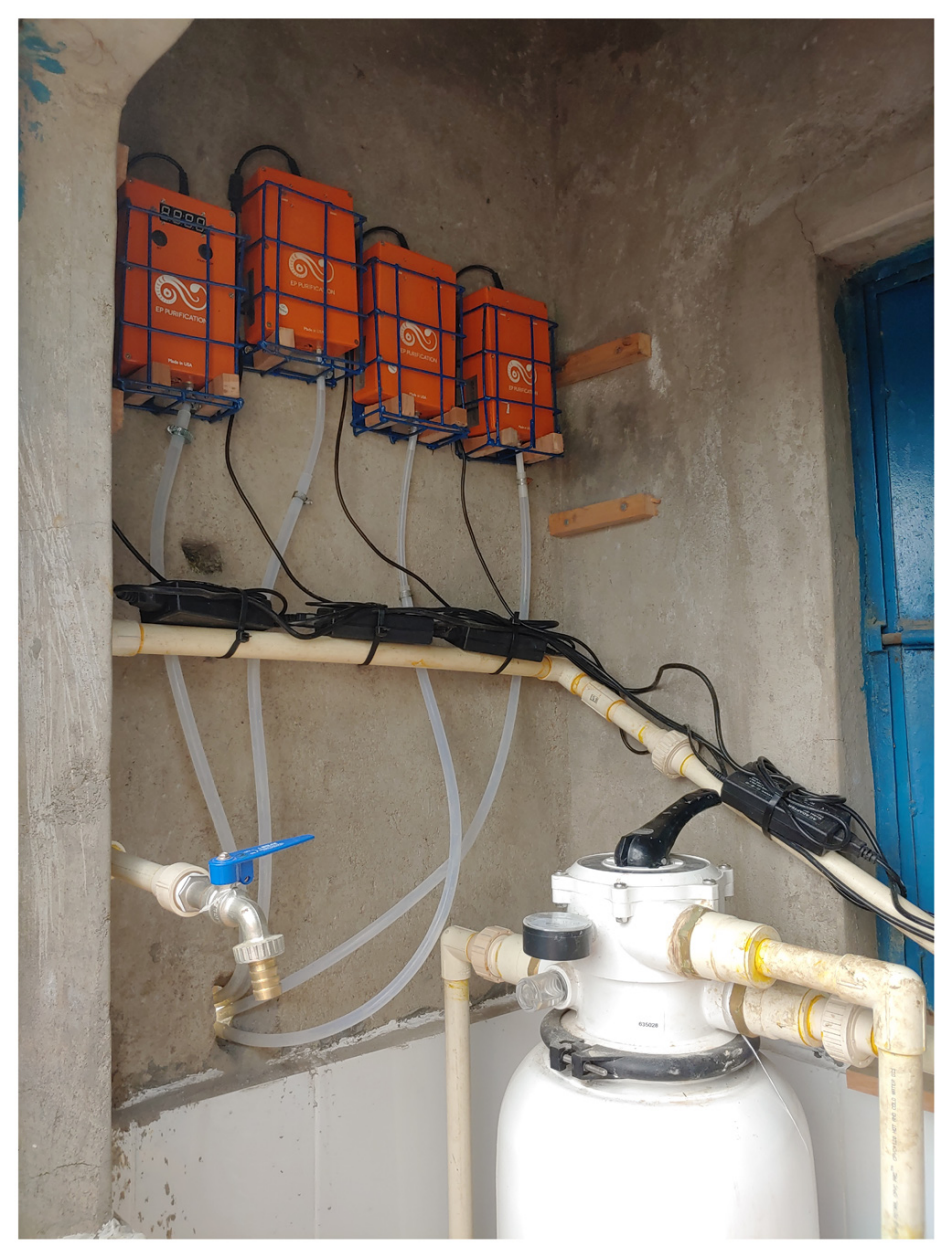
Ozone generators in the utility housing.

***Ozonation.*** Four portable microplasma ozone generators were used (Purelife 1000, EP Purification, Champaign, IL, USA). Each unit weighs 780 grams, and measures 5.4cm × 11.7cm × 18.4 cm, approximately the size of book, and were maintained in the utility housing ( Figure 4). The ozone unit has a miniature diaphragm air pump that draws ambient air at the rate of 2 liters per minute into the array of 250 µm scale (width) channels fabricated in a nanoporous Al _2_O _3_/aluminum chip (reaction volume of 1.9 µL/channel). In the microchannels, oxygen in ambient air (O _2_) is converted to ozone (O _3_) by a high frequency electric field applied across the top and bottom electrodes in the channel. The power consumption of the chip is 10–14 watts per hour. The unit has a built-in rechargeable battery of 7 amperes capacity to operate for 90 minutes if an external power failure were to occur. Each unit produces 0.2–0.35 g of ozone per hour (depending on relative humidity); thus approximately overall 1gram of ozone per hour was produced. The ozone/air mixture flowed from each ozone generator, through tubing and was released into the disinfection tank via an AS150 6-inch fine pore with ¼ inch barb ceramic aerator (diffuser) (Pentair Aquatic Eco-Systems, Apopka, FL, USA). Aerators were positioned at the bottom of the disinfection tank so that air/ozone bubbled up through the 1,000L water tank. A covered vent at the top of the disinfection tank released air/ozone to prevent pressure build-up within the tank. The ozone tubing was Teflon ^®^ and the ozonation tank was PVC because these are considered relatively resistant to oxidation by ozone.

***Electrical supply and controls.*** Initially, the ozonation system and pumps were powered by 240V AC current from electrical outlets. Between Trial 5 and Trial 6, the solarization of the system was completed and from that point forward, the entire system operated exclusively on solar power. Two photovoltaic solar systems, each with its own modules (solar panels) were in place. To power the water treatment system, four roof-mounted 270W Polycrystalline Solar Modules (Yingli Solar) fed current to a Champion 200Ah 12V Sealed Solar Battery. An OPTI SC-PWM 60A 12/24V DC Charge Controller prevents the photovoltaic panels from overcharging battery. An Opti SP Effecto 2000 Hybrid Inverter (Input: 24VDC, Output: 230VAC/50Hz) was used to convert the stored voltage to AC to power pumps in the treatment system and the ozone generators. A separate solar-powered electrical system powered the Pedrollo PKm100 pump 1.1KW that moved water 164 meters from the River Nyando to the treatment system with an elevation gain of 5 meters and a dynamic head of 20 meters. Ten 200W Topray Solar Modules (Shenzhen Topray Solar Co) fed current through a Dayliff 1.5Kw SV2 Sunverter Solar Controller. A Lorentz Pv Disconnect Switch 1000VDC/40A, a C/W earth rod and lightening arrestor were also installed.

### Procedures

***Water sampling.*** Water samples were collected in 120mL IDEXX sampling bottles, which were autoclaved between rounds of sampling. Water samples were collected from a minimum of two sampling ports: river water before it entered the settling tank and from the ozonation tank at 0, 60, 120, 180 and 240 minutes. In three trials, water was also sampled between the balance tank and the sand filter, and again, between the activated carbon filter and the ozonation tank. Water samples were transported within 3–5 hours of collection in a cooler on ice to the Safe Water & AIDS Project (SWAP) laboratory in Kisumu, Kenya.

***Water analysis.*** Turbidity was measured at the SWAP laboratory in Kisumu using the LaMotte 2020we (Chestertown, MD, USA) turbidity meter, which was calibrated daily up to 1,000 NTU. Concentrations of total coliform bacteria and *E. coli* were measured using the Colilert ^®^ (Laboratories, Westbrook, ME, USA) defined substrate culture method, which quantifies bacteria levels in units of MPN per 100mL. This method is approved by the US Environmental Protection Agency for drinking water testing purposes ( USEPA, 2017). Surface water samples were generally, but not always, diluted with 25mL added to 75mL of distilled water and incubated for 24 hours at 35°C. Water samples collected elsewhere in the treatment process were not diluted. The upper limit of quantification of bacteria for undiluted water samples in 2,419.7 MPN/100mL; with the 25mL + 75mL dilution, the upper limit is 9,678.8 MPN/100mL. Generally, but not always, two separate laboratory technicians independently read the Colilert results (number of large and small positive wells).

***Time and place of the trails.*** Data from all trials conducted once the system became operational in September 2019 through to April 2020 are reported here. The decentralized water treatment system was constructed at the site of a defunct water kiosk (0°10'19.7"S 34°55'21.6"E) that had been operated by SWAP in the town of Ahero, in Kisumu County, Kenya. The urban center of Ahero is located 22 km east of the city of Kisumu and has a population of 11,801 ( Kenya National Bureau of Statistics, 2019). The River Nyando flows through the center of Ahero.

***Independent laboratory analyses.*** Between Trials 4 and 5, the Water Resources Authority, and separately, the Kenya Bureau of Standards independently analyzed water samples and evaluated whether the finished water met World Health Organization (WHO) and Kenya’s Drinking Water Standards.

***Data analysis.*** Turbidity, total coliform, and *E. coli* measures for each stage in the treatment process were analyzed for normality using the Kolmogorov-Smirnov test. Bacteria levels were not distributed normally and are summarized as the median and interquartile range (25 ^th^–75 ^th ^percentile). Log reduction values (LRV) of bacteria were calculated as log10(raw water concentration)-log10(ozonated water concentration). Because log10 of zero is not defined, a concentration of 0.1 MPN/100mL was used in the calculation of LRV no *E. coli* were detected in a sample. Data were analyzed using MS Excel and SAS version 9.4 (SAS Institute, Cary, N.C., USA).

***Metals analysis.*** While the disinfection system was undergoing initial testing (before Trial 1 was conducted) aluminum, manganese and iron were tested. Finished water samples were analyzed using the HACH Model DR 3900 Laboratory Spectrophotometer. Reagents and methods used were HACH method 8012 for aluminum, Method 8034 for manganese, and FerroVer ^®^ for iron.

## Results

Once the decentralized system was built and tested, nine trails were conducted to evaluate system performance. Table 1 describes water quality, as measured by bacteria concentration and turbidity, at baseline (raw water), after flocculation and filtration (ozonation t=0), and following ozonation at several timepoints out to t=240 minutes. It is clear that substantial reductions in *E. coli*, total coliforms, and turbidity occurred, mainly as a result of flocculation and filtration. Table 2 presents LRV for *E. coli* showing median LRV reductions of approximately 3.8 Sensitivity analysis was conducted of the *E. coli* LRV, in which 0.5 rather than 0.1 was used as the *E. coli* concentration for calculating LRV in cases of non-detects. The median *E. coli* LRV using that more conservative approach was 3.38.

**Table 1.** Water quality at baseline (raw water), after filtration and flocculation (ozonation T=0), and during ozonation. MPN, most probable number; NTU, nephelometric turbidity units; T, duration of ozonation, in minutes.

Water	N	E. coli median MPN/100mL (25 ^th^-75th Percentile)	Total coliforms median MPN/100mL (25 ^th^-75th Percentile)	N	Turbidity median (NTU) (25 ^th^-75th Percentile)
**Raw**	34	2,419.7 (2,419.7, 2,419.7)	2,419.7 (2,419.7, 2,419.7)	31	283.3 (201.7, 863.5)
**Ozonation T=0**	46	10.9 (3, 67.7)	1016.9 (94.1, 2419.7)	44	3.8 (2.2, 4.7)
**Ozonation T=60**	30	2.1 (1, 17.1)	227.4 (14.4, 2419.7)	29	2.8 (2.3, 4.2)
**Ozonation T=120**	46	1 (0, 8.6)	25.05 (4.1, 1553.1)	44	3.4 (2.4, 4.5)
**Ozonation T=180**	46	0 (0, 1)	4.6 (0, 235.9)	41	3.2 (2.1, 4.4)
**Ozonation T=240**	11	1 (0, 1)	2.0 (1, 39.5)	13	2.7 (2.0, 4.2)

**Table 2.** Log removal values of *E. coli* and total coliforms, by treatment step. N =46.

Indicator bacteria	Treatment step	Log reduction values Median (25th-75 ^th^ Percentile)
Total coliforms	Flocculation and filtration	0.60 (0.23-1.64)
	Ozonation for 180 minutes	1.21 (0.71-2.29)
	Flocculation, filtration, ozonation	2.95 (1.24-3.73)
*E. coli*	Flocculation and filtration	2.25 (1.57-3.10)
	Ozonation for 180 minutes	0.93 (0.12-2.49)
	Flocculation, filtration, ozonation	3.69 7(3.19- 4.57)

### Independent laboratory testing

The Kenya Bureau of Standards analyzed finished water from the decentralized system and reported that *E. coli* and coliform concentrations were reported as 0 colony forming units/100mL. The Water Resources Authority tested River Nyando water and found that turbidity was >1,000 NTU (WHO and Kenyan standard: <5 NTU). *E. coli* and coliform bacteria were “too numerous to count.” Finished water met WHO and Kenyan drinking water standards: turbidity was 4.5 NTU, and 0 CFU/100mL of *E. coli* and coliforms were detected.

Metals analysis of finished water (prior to Trial 1) showed concentrations of metals that were well within Kenyan Drinking Water Standards: aluminum: 0.01mg/L, iron 0.005 mg/L, and manganese 0.7 mg/L.

## Discussion

We observed nearly 4-log orders of E. coli removal using the decentralized, solar powered water disinfection system. The use of solar energy for drinking water treatment has been promoted, in part because regions of the world with little access to safe drinking water tend be in equatorial regions, where sunlight is plentiful ( Chu *et al.*, 2019; Pichel *et al.*, 2019). To the best of our knowledge, this is the first report of a decentralized drinking water treatment system that uses ozone disinfection. The water treatment system was able to consistently process extremely turbid water with high levels of fecal indicator bacteria, and to produce 1,000 L of water with turbidity levels and *E. coli* levels that met WHO drinking water guideline values ( WHO, 2017). WHO’s Technology Non-Specific Harmonized Testing Protocol established performance standards for POU methods ( World Health Organization, 2014). The evaluation process should assess performance with “pre-treatment” concentration of bacteria of 100,000/100mL and turbidity is set at 40±10 NTU. In our ‘real-world’ setting, the water sample dilution method resulted in a maximal measurable bacterial concentration of 9,682.8. Thus, the observed LRVs likely underestimate actual LRVs. The extremely high pre-treatment water turbidity exceeded by a wide margin WHO test protocol requirements.

It should be noted that very large reductions in turbidity and fecal indicator bacteria took place prior to ozonation. Although bacterial concentrations decreased substantially, viruses may persist following filtration, and a disinfection step should further decrease concentrations of viruses. We have demonstrated previously that ozonation eliminated coliphage viruses from sewage samples ( Dorevitch *et al.*, 2020), though chlorination is certainly effective in reducing viruses ( WHO, 2017). *Cryptosporidium spp*. oocysts are relatively chlorine-resistant ( WHO, 2017), though they are reduced significantly by ozonation ( Donofrio *et al.*, 2013). Thus, the ozonation step following filtration in the system we evaluated should have reduced waterborne virus and *Cryptosporidium spp.* concentrations. Though chlorination following filtration would also be expected to reduce bacterial and viral concentrations as a disinfection step, the taste of chlorinated water is often unacceptable in low- and middle-income communities ( Roma *et al.*, 2014; Rosa & Clasen, 2017; Rothstein *et al.*, 2015). By contrast, in a small study of POU ozonation in Kenya, we found the taste of the finished water to be acceptable ( Dorevitch *et al.*, 2020).

Ozone disinfection has been largely limited to Europe and North America, in part because of the high costs of constructing and powering centralized ozone generation on a large scale ( Loeb *et al.*, 2012; Mundy *et al.*, 2018). A disadvantage of ozonation is that, unlike chlorination, it does not leave a residual level of a disinfectant in treated water. For that reason, education about safe water storage, such as the use of improved vessel containers with tap, lid and narrow neck, will be important when providing disinfected drinking water from the Ahero system. Ozonation of bromide can produce bromates ( von Gunten, 2003; Yang *et al.*, 2019), which are possible carcinogens ( IARC, 1999). For that reason, ozonation of groundwater should be done only after ensuring that bromide is not present.

The role of all-solar decentralized ozonation systems in the global effort toward achieving UN Sustainable Development Goal target 6.1 (safe and accessible drinking water for all by 2030) remains to be determined. Membrane filtration methods have been the focus of decentralized water treatment studies ( Francis *et al.*, 2016; Huttinger *et al.*, 2015; Sima *et al.*, 2012). Before systems, such as the one we described, could be widely deployed, research will be needed to determine the relative effectiveness, costs, impacts on health, and community acceptance of the ozonation and membrane filtration. By operating the system on solar power, this approach does not release particulate matter, carbon monoxide, and other air pollutants. This addresses concerns that arise when water treatment and air quality are not considered jointly ( Clasen & Smith, 2019).

The cost of materials and construction was approximately $24,000, though several tanks and kiosk structures were available at the defunct site when work began. Though in the trials reported here we treated 1,000L per day, two treatment cycles per day can be run, doubling output to 2,000 L with only a marginal increase in cost (primarily the time of the system operator and the cost of alum). The ozonation units are modular, and with several ozonation tanks, each linked to larger ozone generators, the cost per cubic meter of water produced can be decreased substantially. By assembling ozone generators and other system elements in Kenya, costs would decrease further. If future sites like this are developed, systems can be simplified, bringing down costs. Two modifications we intend to make are the elimination of the “balance tank” and directing ozone exhaust from the disinfection tank back to the settling tank for water pre-treatment.

The findings of this research are subject to several limitations. The number of trials was relatively small, and they took place over a six-month period. It is possible that system performance will decrease over longer time periods. The dilution of raw water samples was 25mL in 75mL of distilled water, and dilution was only done in the first four trials, limiting the upper limit of bacterial quantification. As a result, the reported LRVs likely under-estimate actual system performance. The research did include measures of ozone concentration in the disinfection process, though this has been measured during smaller scale laboratory trials, with concentrations of 0.28–0.40ppm ( Dorevitch *et al.*, 2020). This research did not address community perspectives. However, the earlier work of POU ozonation (without turbidity reduction) found the taste of the water – but not the cloudiness of the water – to be acceptable.

## Conclusion

Based on the substantial reduction in enteric bacteria and turbidity, we conclude that a solar-powered decentralized water system with microplasma ozone generating units can effectively treat highly contaminated surface waters. We believe this approach has several favorable features: 1) it is entirely solar-powered, 2) it is scalable, 3) it is effective in treating highly turbid water, 4) it is relatively simple to operate with training, and 5) the capital costs are small compared to those of a centralized treatment system. Further research will be needed to optimize this approach and characterize its limits.

## Data availability

### Underlying data

Harvard Dataverse: Decentralized solar-powered drinking water ozonation in Western Kenya: An evaluation of disinfection efficacy, https://dataverse.harvard.edu/dataset.xhtml?persistentId=doi:10.7910/DVN/0SD71Q ( Dorevitch, 2020).

Data are available under the terms of the Creative Commons Zero "No rights reserved" data waiver (CC0 1.0 Public domain dedication).
