# Supplementary material for: Decentralized solar-powered drinking water ozonation in Western Kenya: an evaluation of disinfection efficacy
Source: Gates Open Res. 2020 Oct 15;4:56. Originally published 2020 May 29. [Version 2] doi: 10.12688/gatesopenres.13138.2 (PMC7644876; doi:10.12688/gatesopenres.13138.2)
Supplement: Peer review history

# Peer review: Reviewer response for version 2

**DOI:** 10.21956/gatesopenres.14404.r29817

**Published:** 2020-11-27

**Authors:** Liane Nakada

**Affiliations:** 1 School of Civil Engineering, Architecture and Urban Design - Department of Environment and Sanitation, University of Campinas, São Paulo, Brazil

The authors revised the manuscript according to the reviewers' comments. Furthermore, more trials were included in this second version.

Is the work clearly and accurately presented and does it cite the current literature?

Partly

If applicable, is the statistical analysis and its interpretation appropriate?

Yes

Are all the source data underlying the results available to ensure full reproducibility?

Yes

Is the study design appropriate and is the work technically sound?

Partly

Are the conclusions drawn adequately supported by the results?

Partly

Are sufficient details of methods and analysis provided to allow replication by others?

Partly

Reviewer Expertise:

My research interests are focused on water treatment, giardia inactivation, ozonation, solar-driven disinfection, and rainwater harvesting.

I confirm that I have read this submission and believe that I have an appropriate level of expertise to confirm that it is of an acceptable scientific standard.

# Peer review: Reviewer response for version 2

**DOI:** 10.21956/gatesopenres.14404.r29816

**Published:** 2020-11-05

**Authors:** Urmila Brighu, Ashish Tambi

**Affiliations:** 1 Department of Civil Engineering, Malaviya National Institute of Technology Jaipur, Jaipur, Rajasthan, India; 2 Department of Environmental Sciences, IIS (Deemed to be University), Jaipur, Rajasthan, India

We have gone through the revisions and found them to be appropriate.

Is the work clearly and accurately presented and does it cite the current literature?

Yes

If applicable, is the statistical analysis and its interpretation appropriate?

No

Are all the source data underlying the results available to ensure full reproducibility?

Partly

Is the study design appropriate and is the work technically sound?

Partly

Are the conclusions drawn adequately supported by the results?

Partly

Are sufficient details of methods and analysis provided to allow replication by others?

No

Reviewer Expertise:

NA

We confirm that we have read this submission and believe that we have an appropriate level of expertise to confirm that it is of an acceptable scientific standard.

# Peer review: Reviewer response for version 2

**DOI:** 10.21956/gatesopenres.14404.r29815

**Published:** 2020-10-26

**Authors:** Geremew Sahilu

**Affiliations:** 1 Addis Ababa Institute of Technology, Addis Ababa University, Addis Ababa, Ethiopia

I have gone through the revised version and hereby changed the status to Approved based on the following modifications:

1. Increased number of trials;
2. Dimensions incase it is required for replication and;
3. Explanation why ozonation after coagulation and filtration while these have changed turbidity and fecal indicators level significantly.

Is the work clearly and accurately presented and does it cite the current literature?

Yes

If applicable, is the statistical analysis and its interpretation appropriate?

Yes

Are all the source data underlying the results available to ensure full reproducibility?

Partly

Is the study design appropriate and is the work technically sound?

Partly

Are the conclusions drawn adequately supported by the results?

Partly

Are sufficient details of methods and analysis provided to allow replication by others?

No

Reviewer Expertise:

NA

I confirm that I have read this submission and believe that I have an appropriate level of expertise to confirm that it is of an acceptable scientific standard.

# Peer review: Reviewer response for version 1

**DOI:** 10.21956/gatesopenres.14326.r29432

**Published:** 2020-09-10

**Authors:** Urmila Brighu, Ashish Tambi

**Affiliations:** 1 Department of Civil Engineering, Malaviya National Institute of Technology Jaipur, Jaipur, Rajasthan, India; 2 Department of Environmental Sciences, IIS (Deemed to be University), Jaipur, Rajasthan, India

1. The primary goal of this research is to evaluate the efficiency of decentralized solar-powered drinking water treatment using Ozone disinfection. Ozonation as a disinfectant practice is well established and documented. It was not very clear as to what the whole paper is about or what is the novelty when the reliability of ozone production affecting the dosing and retention times (CT) in the context of decentralized solar powered ozonator were not discussed. Also, as shown in Table 1, the pre-ozonation treatment (flocculation process) has already reduced the *E.coli* concentration to zero and therefore, the effect of ozonation is not clear. 
2. The water treatment system defined in the text is not matching with the flow chart in Figure 1. “Following ozonation (described next), the water flows through the So-Safe Triple Multibody 10” Filter (8 bar) with an activated carbon and two sediment cartridges.”
3. It was not very clear as to why turbidity should increase after ozonation as indicated in Table 1.
4. “However, because we have demonstrated significant reduction in viral indicators using ozone, and because the taste of the finished water has been found to be very good by users, we intend to continue using ozone as the disinfection method ( Dorevitch *et al.*, 2020)”
  Could not access the above reference.
5. The methods used in this study are not presented clearly.
6. Brief description of trials including the ozone dosage and sampling points in Table no. 2 would further help in understanding the actual log reductions of total coliforms and *E.coli*..
7. The number of trials is less (only 9) which is not sufficient enough for detailed assessment of the performance of the system.
8. Since ozone as a disinfectant has a major disadvantage of not leaving any residual disinfectant to guard against the possible contamination during storage, a detailed cost analysis and its comparison with other methods like UV disinfection including arrangements for residual disinfectant and chlorination method can further improve this manuscript especially when it is proposed for rural settings.

Is the work clearly and accurately presented and does it cite the current literature?

Yes

If applicable, is the statistical analysis and its interpretation appropriate?

No

Are all the source data underlying the results available to ensure full reproducibility?

Partly

Is the study design appropriate and is the work technically sound?

Partly

Are the conclusions drawn adequately supported by the results?

Partly

Are sufficient details of methods and analysis provided to allow replication by others?

No

Reviewer Expertise:

Water Supply, Water and Sanitation, Waste Water Treatment

We confirm that we have read this submission and believe that we have an appropriate level of expertise to state that we do not consider it to be of an acceptable scientific standard, for reasons outlined above.

# Response

**Published:** 2020-10-01

**Authors:** Samuel Dorevitch

The authors wish to thank Prof. Brighu for the very helpful feedback. Responses to those comments follow.

**1. The primary goal of this research is to evaluate the efficiency of decentralized solar-powered drinking water treatment using Ozone disinfection. Ozonation as a disinfectant practice is well established and documented.**

We agree with Prof. Brighu that ozonation is certainly well-established and documented. However, the ozonation of drinking water and wastewater is conducted in large, centralized, treatment facilities, and generally produced using 100% oxygen or  dehumidified air. We are aware of no prior studies of the production, on-site, of ozone using ambient air, nor are we aware of studies of decentralized water disinfection using solar power. We have revised the end of the ‘Introduction’ section to better convey the novelty of the way that ozone is generated and used.

**2. It was not very clear as to what the whole paper is about or what is the novelty when the reliability of ozone production affecting the dosing and retention times (CT) in the context of decentralized solar powered ozonator were not discussed. Also, as shown in Table 1, the pre-ozonation treatment (flocculation process) has already reduced the *E.coli* concentration to zero and therefore, the effect of ozonation is not clear.**

We did not measure aqueous ozone in Kenya. We have previously reported that in the laboratory in the US, we did measure concentrations of aqueous ozone (0.28-0.40ppm) produced using the (microplasma, small scale) process. This has been added to the last paragraph of the Discussion section.

**3. The water treatment system defined in the text is not matching with the flow chart in Figure 1. “Following ozonation (described next), the water flows through the So-Safe Triple Multibody 10” Filter (8 bar) with an activated carbon and two sediment cartridges.”**

Thank you for pointing this out. Figure 1 in Version 2 of the manuscript includes the post-ozonation filters.

**4. It was not very clear as to why turbidity should increase after ozonation as indicated in Table 1.**

In version 1 of the manuscript, only 4 measurements of turbidity at t=240 minutes were available. In version 2, with a much larger number of observations, no increase in turbidity over time is apparent.

**5. “However, because we have demonstrated significant reduction in viral indicators using ozone, and because the taste of the finished water has been found to be very good by users, we intend to continue using ozone as the disinfection method ( Dorevitch *et al.*, 2020** **)” Could not access the above reference.**

A link to the above-reference publication has been added to the list of citations.

**6. The methods used in this study are not presented clearly.**

We have revised the ‘Methods’ section with the aim of improving clarity.

**7. Brief description of trials including the ozone dosage and sampling points in Table no. 2 would further help in understanding the actual log reductions of total coliforms and *E.coli*..**

Table 2 now makes clear that LRVs are based on initial (raw) water and final (post-ozonation but pre-final filtration) measurements. As noted, ozone concentrations were not measured in this study.  We note in the Methods section that each ozone generator produced approximately 0.25gm ozone/hour, so that the four ozone generators operating simultaneously produced approximately 1g ozone/hours.  

**8. The number of trials is less (only 9) which is not sufficient enough for detailed assessment of the performance of the system.**

Since the time that Version 1 of the paper was submitted, we have conducted 38 additional trials. The results of all 47 trials are summarized in Version 2 of the manuscript, and the data from those trials have been uploaded to the Harvard Dataverse.

**9. Since ozone as a disinfectant has a major disadvantage of not leaving any residual disinfectant to guard against the possible contamination during storage, a detailed cost analysis and its comparison with other methods like UV disinfection including arrangements for residual disinfectant and chlorination method can further improve this manuscript especially when it is proposed for rural settings.**

We note in the Discussion section that the absence of a residual disinfectant is a disadvantage of ozonation. However, ozonation also has advantages relative to chlorination, such as efficacy against Cryptosporidium oocysts and the lack of a ‘chemical taste’ in the finished water. This is also noted in “Discussion.”

# Peer review: Reviewer response for version 1

**DOI:** 10.21956/gatesopenres.14326.r29434

**Published:** 2020-09-08

**Authors:** Liane Nakada

**Affiliations:** 1 School of Civil Engineering, Architecture and Urban Design - Department of Environment and Sanitation, University of Campinas, São Paulo, Brazil

This manuscript describes an interesting point-of-use drinking water treatment system using solar-powered ozonation. Please find below my suggestions to improve the overall quality of the manuscript:

- It is important to provide detailed information regarding the treatment system:
  - Why 935 g aluminum sulfate? 
  - What is the final concentration of aluminum sulfate in mg/L considering raw water flow? 
  - gm is not correct, the correct form is g (international system of units).
  - Why glass media? Why not sand? The reasons may include efficiency, backwashing, and cost.
- Turbidity of harvested rainwater is not always low. Please consider a first flush device if treating rainwater.  
- Nine different trials were conducted. A summary of these trials indicating ozone dosage (mg/L), sampling points, and analyzed parameters would facilitate reading.
- Table 1 is confusing. It would be better to present the results by trial, and not by sampling point.
- The main conclusion is focused on the reduction of enteric bacteria following ozonation, however, the results show that E.coli standard is achieved following flocculation, therefore, credit should not be granted to ozonation.    

Is the work clearly and accurately presented and does it cite the current literature?

Partly

If applicable, is the statistical analysis and its interpretation appropriate?

Yes

Are all the source data underlying the results available to ensure full reproducibility?

Yes

Is the study design appropriate and is the work technically sound?

Partly

Are the conclusions drawn adequately supported by the results?

Partly

Are sufficient details of methods and analysis provided to allow replication by others?

Partly

Reviewer Expertise:

My research interests are focused on water treatment, giardia inactivation, ozonation, solar-driven disinfection, and rainwater harvesting.

I confirm that I have read this submission and believe that I have an appropriate level of expertise to confirm that it is of an acceptable scientific standard, however I have significant reservations, as outlined above.

# Response

**Published:** 2020-10-01

**Authors:** Samuel Dorevitch

Many thanks to Professor Nakada for the constructive comments, which have been addressed in the revised manuscript (Version 2).

The reference and rationale for the alum dose been added to the first paragraph of the Methods section.

The concentration of alum was not measured in the treatment process, though as noted at the end of the "Results" section, in initial trials, aluminum concentrations in finished water were 0.01mg/L.  

The following rationale and a reference for the use of glass media for filtration has been added to the 'Methods" section:

Glass media is generally less costly than sand and in a laboratory evaluation of sand and glass filtration, was found to provide more rapid filtration without a loss of efficacy (Healy *et al*., 2010).

In that paragraph, we no longer refer to rainwater as 'low turbidity.'

The abbreviation 'g' has replaced 'gm' throughout the manuscript.

We agree with the reviewer that flocculation and filtration (without disinfection) certainly did reduce E. coli concentrations substantially.  The second paragraph of the 'Discussion' section in Version 2 of the manuscript now reads:

It should be noted that very large reductions in turbidity and fecal indicator bacteria took place prior to ozonation. Although bacterial concentrations decreased substantially, viruses may persist following filtration, and a disinfection step should further decrease concentrations of viruses.  We have demonstrated previously that ozonation eliminated coliphage viruses from sewage samples ( Dorevitch *et al.*, 2020), though chlorination is certainly effective in reducing viruses  (WHO, 2017). *Cryptosporidium spp.* oocysts are relatively chlorine-resistant ( WHO, 2017), though they are reduced significantly by ozonation (Donofrio *et al*., 2013). Thus, the ozonation step following filtration in the system we evaluated should have reduced waterborne virus and *Cryptosporidium spp.* concentrations.  Though chlorination following filtration would also be expected to reduce bacterial and viral concentrations as a disinfection step, the taste of chlorinated water is often unacceptable in low- and middle-income communities  (Roma *et al.*, 2014; Rosa & Clasen, 2017; Rothstein *et al.*, 2015 ). By contrast, in a small study of  POU ozonation in Kenya, we found the taste of the finished water to be acceptable ( Dorevitch *et al.*, 2020).

The reviewer pointed out the need to clarify information in Tables 1 and 2.

The solar-powered treatment system has continued operation in the  months since Version 1 of the manuscript was submitted and data are available from 47, rather than 9 trials. We believe that Tables 1 and 2, which include data from those trials, are clearer.

# Peer review: Reviewer response for version 1

**DOI:** 10.21956/gatesopenres.14326.r29232

**Published:** 2020-08-24

**Authors:** Geremew Sahilu

**Affiliations:** 1 Addis Ababa Institute of Technology, Addis Ababa University, Addis Ababa, Ethiopia

In general the research shows the application of solar powered decentralized water treatment utilizing ozone. However, the method is not clearly presented since there are no clear dimensions except volumes. This makes it difficult to replicate. Pre-ozone treatments have already achieved the requirement for E. coli and turbidity hence it is not clear what the effect of ozone treatment is. Moreover general information such as specific cost of treatment per cubic meter is not presented to compare it with other methods.

Is the work clearly and accurately presented and does it cite the current literature?

Yes

If applicable, is the statistical analysis and its interpretation appropriate?

Yes

Are all the source data underlying the results available to ensure full reproducibility?

Partly

Is the study design appropriate and is the work technically sound?

Partly

Are the conclusions drawn adequately supported by the results?

Partly

Are sufficient details of methods and analysis provided to allow replication by others?

No

Reviewer Expertise:

My area of research is water supply, water treatment, sanitation, urban drainage in general water supply and sanitation thematic areas.

I confirm that I have read this submission and believe that I have an appropriate level of expertise to confirm that it is of an acceptable scientific standard, however I have significant reservations, as outlined above.

# Response

**Published:** 2020-10-01

**Authors:** Samuel Dorevitch

Many thanks to Professor G. Sahilu for the helpful feedback. 

Regarding system specifications: we added dimensions of the system to the first paragraph of the "Methods" section, which contains details  regarding treatment times, alum dosing, and the product/manufacturer information of system components. We did not measure aqueous ozone concentrations, which is  noted as a limitation of the study.

We agree that the coagulation, flocculation, and filtration steps resulted in dramatic improvements in water quality before the ozonation step.  The second paragraph of the "Discussion" section now notes the following:

It should be noted that very large reductions in turbidity and fecal indicator bacteria took place prior to ozonation. Although bacterial concentrations decreased substantially, viruses may persist following filtration, and a disinfection step should further decrease concentrations of viruses.  We have demonstrated previously that ozonation eliminated coliphage viruses from sewage samples ( Dorevitch *et al.*, 2020), though chlorination is certainly effective in reducing viruses  (WHO, 2017). *Cryptosporidium spp.* oocysts are relatively chlorine-resistant ( WHO, 2017), though they are reduced significantly by ozonation (Donofrio *et al*., 2013). Thus, the ozonation step following filtration in the system we evaluated should have reduced waterborne virus and *Cryptosporidium spp.* concentrations.  Though chlorination following filtration would also be expected to reduce bacterial and viral concentrations as a disinfection step, the taste of chlorinated water is often unacceptable in low- and middle-income communities  (Roma *et al.*, 2014; Rosa & Clasen, 2017; Rothstein *et al.*, 2015 ). By contrast, in a small study of  POU ozonation in Kenya, we found the taste of the finished water to be acceptable ( Dorevitch *et al.*, 2020).
